# Modeling of COVID-19 propagation with compartment models

**DOI:** 10.1007/s00591-021-00312-9

**Published:** 2021-11-03

**Authors:** Günter Bärwolff

**Affiliations:** Inst. f. Math., Technische Universität Berlin, Str. des 17. Juni 136, 10623 Berlin, Germany

**Keywords:** Mathematical epidemiology, SIR-type model, Model parameter estimation, COVID-19/SARS-CoV‑2

## Abstract

The current pandemic is a great challenge for several research areas. In addition to virology research, mathematical models and simulations can be a valuable contribution to the understanding of the dynamics of the pandemic and can give recommendations to both physicians and politicians. In this paper we give an overview about mathematical models to describe the pandemic by differential equations. As a matter of principle the historic origin of the epidemic growth models will be remembered. Moreover we discuss models for the actual pandemic of 2020/2021. This will be done based on actual data of people infected with COVID-19 from the European Centre for Disease Prevention and Control (ECDC), input parameters of mathematical models will be determined and applied. These parameters will be estimated for the UK, Italy, Spain, and Germany and used in a *SIR*-type model. As a basis for the model’s calibration, the initial exponential growth phase of the COVID-19 pandemic in the named countries is used. Strategies for the commencing and ending of social and economic shutdown measures are discussed. To respect heterogeneity of the people density in the different federal states of Germany diffusion effects are considered.

## Introduction

**Fig. 1:**
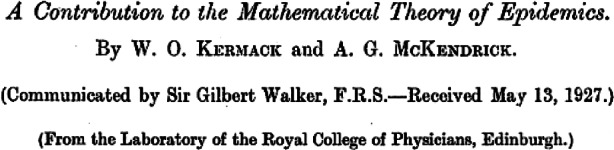
Original title, facsimile of [26]

The origin of the often used *SIR*-type models is given with the fundamental paper of Kermack and McKendrick ([26]; Fig. 1).

Their model was applied on pandemics at the beginning of the 20th century, especially on the Spanish flu pandemic. The application of the model led to some important recommendations of prevention to combat the transmissions of pandemic viruses.

**Fig. 2:**
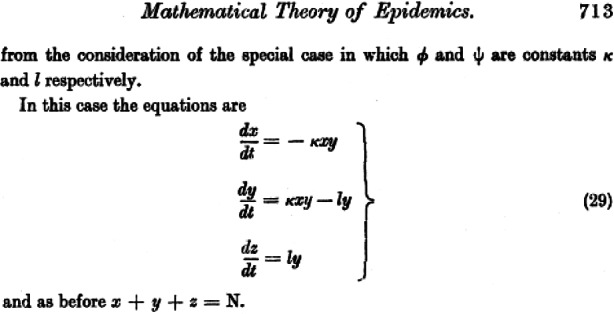
Original ode-system, facsimile of [26]

Fig. 2 shows the part of the Kermack and McKendrick paper where they wrote down the relevant ordinary differential equations for the development of the susceptible ($S$), infected ($I$), and recovered/removed^1^ ($R$) people in a pandemic.

The dynamic development of sub-populations of susceptible, infected and removed people in a certain region, for example, the population of a country or a part of a federation, is the aim of the modeling. First deterministic models are discussed. These are simple but effective for describing the progression of the pandemic. They are able to fit the description of the average infection dynamics in macroscopic sub-populations only.

Given the fact that there is a lot of controversy in the applied math community it is important to mention that the discussed *SIR*-type models are an approximation of the pandemic. They do not claim the absolute and precise description of the COVID-19 pandemic. But we think that these models will be a valuable contribution to the dynamics of the epidemic.

The description of the pandemic with a finer resolution of the pandemic is possible with stochastic agent-based models but this is not considered and discussed in this paper. Some interesting results in this can be found in [27, 34, 43].

More complex deterministic models based on the original *SIR* model are also discussed. These include sub-populations other than $S$, $I$, and $R$ (see [24, 32]), but these models have dynamic properties similar to those of the basic *SIR* model. These models are presented and also discussed.

Beside the discussion of the undisturbed pandemic, without any pharmaceutical or non-pharmaceutical intervention, we look for possibilities and conditions to stop or reduce the spread of the viruses. Suggestions about favorable points in time at which to commence with lockdown measures based on the acceleration rate of infections are also discussed.

It is necessary to remark that the considered *SIR* model is not able to describe the full asymptotic behavior of a pandemic, as is done in [40]. In addition, the role of super-spreaders, investigated in [35] and [8], cannot be described with the basic macroscopic *SIR* model^2^. Beside these limitations the *SIR* model describes the mean pandemic behavior in an acceptable quantity and quality.

## The mathematical *SIR* model

First, we note one important presupposition for the model. We suppose that the distribution of the included sub-populations is equal, i.e., the density is approximately constant. This is a very strict supposition, but this is acceptable, for example, for cities and congested urban areas like New York or the Ruhr area in Germany. At the beginning of the pandemic, exponential growth of the number of infected people is supposed.

In the so-called *SIR* model of Kermack and McKendrick [26], $I$ denotes the infected people, $S$ denotes the susceptible people, and $R$ denotes the recovered or removed people.^3^ It is a deterministic model. Here we constrain the investigations to the species $I$, $S$, and $R$ only. The dynamics of infections and recoveries can be approximated by the following system of ordinary differential equations:

1 $$\begin{aligned}\frac{dS}{dt} & = & -\beta\frac{S}{N}I\end{aligned}$$

2 $$\begin{aligned}\frac{dI}{dt} & = & \beta\frac{S}{N}I-\gamma I\end{aligned}$$

3 $$\begin{aligned}\frac{dR}{dt} & = & \gamma I\;.\end{aligned}$$

$\beta$ represents the number of others that one infected person encounters per unit time (per day). $\gamma$ is the reciprocal value of the typical time from infection to recovery. $N$ is the total number of people involved in the epidemic disease, and $N=S+I+R$ and it follows from the equation that $N$ is constant. It was supposed that an infected person of compartment $I$ is immediately infectious. This supposition was given up later in more complex models. The phenomenon of a delayed occurrence of an infection or the ability to transmit the virus can also described with delay differential equations (see for example [31], [19]).

The currently available empirical data [14] suggest that the corona virus infection typically lasts for about 14 days. This means that $\gamma=1/14\approx 0.07$. The choice of $\beta$ is more complicated and will be considered in the following.

The equation system (1)–(3) belongs to the mathematical category of dynamical systems.

## The estimation of $\beta$ based on real data

We use the European Centre for Disease Prevention and Control [15] as data for the COVID-19 infected people for the period from January 31, 2019 to April 8, 2020.

At the beginning of the pandemic the quotient $S/N$ is nearly equal to 1. Also, at the early stage no-one has yet recovered. Thus we can describe the early regime by the ordinary differential equation

$$\frac{dI}{dt}=\beta I$$

with the solution

4 $$I(t)=I_{0}\exp(\beta t)\;.$$

The logarithm of (4) leads to

$$\log I(t)=\log I_{0}+\beta t\;.$$

Based on the table $(t_{j},\,\log I_{j}),\,j=1,\dots,k$, of logarithms of the infected people versus time the functional

5 $$L(I_{0},\beta)=\sum_{j=1}^{k}[\log I_{0}+\beta t_{j}-\log I_{j}]^{2}\;,$$

is to minimize. The ansatz (5) is very popular because it has a very simple structure, leads to a linear regression problem and is often used in medical science.

**Fig. 3:**
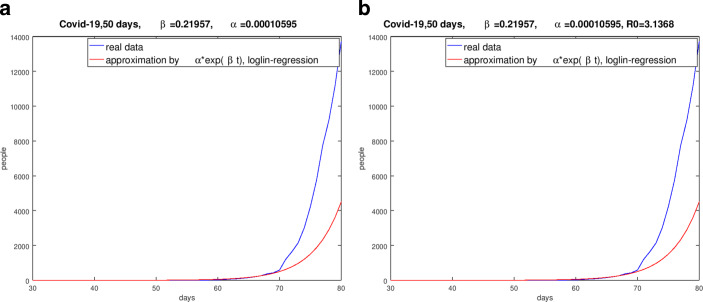
Regime of the pandemic, $\beta$-value from the logarithmic-linear regression. **(a)** Result for Spain (January 31, 2020 to March 20, 2020), **(b)** Result of the UK (January 30, 2020 to March 20, 2020)

Fig. 3 shows the results for the same periods as above for Spain and the UK.

Fig. 3 shows that the logarithmic-linear regression implies unsatisfactory results of the approximation of the real data by the ansatz (4). It must be said that evaluated $\beta$-values are related to the stated period.

Instead of the above used table of logarithmic values the table $(t_{j},\,I_{j}),\,j=1,\dots,k$, is used with the aim of a better approximation. We are looking for periods from the beginning of the pandemic in the spreadsheets of infected people per day where the run can be described by a function of type (4).

Choosing a period $[t_{1},t_{k}]$ for a certain country we search for the minimum of the functional

$$F(I_{0},\beta)=\sum_{j=1}^{k}[I_{0}\exp(\beta t_{j})-I_{j}]^{2}\;,$$

i.e.

6 $$\min_{(I_{0},\beta)\in\mathbb{R}^{2}}F(I_{0},\beta)\;.$$

**Fig. 4:**
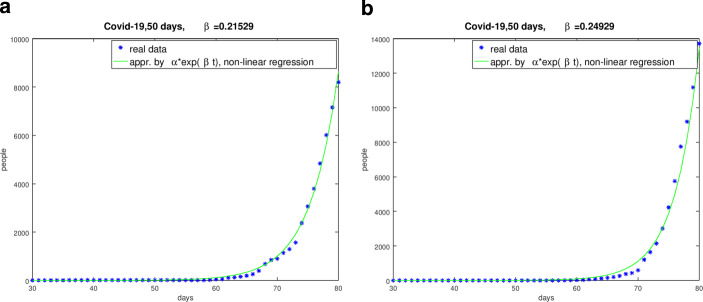
Comparison of the real data and the curves with the evaluated parameter $\beta$ from January 31, 2020 to March 20, 2020. **(a)** German regime from January 31, 2020 to March 20, 2020, **(b)** Spanish regime from January 31, 2020 to March 20, 2020

We solved this non-linear minimum problem with the damped Gauss-Newton method. The results of the logarithmic-linear method for $\beta$ and $\alpha$ as discussed above proved as good start iterations for the Gauss-Newton method. We found the subsequent results for the considered countries. Thereby we choose such periods for the countries with the aim to approximate the infection succession in a good quality. Fig. 4 shows the graphs and the evaluated parameter for Germany and Spain.

We found some information on the parameters in Italy in [16], for example $\beta=0.25$, and we presume that this is a result of the logarithmic-linear regression by the Italian health administration.

A deeper look at the real data shows that the exponential behavior of the dynamic of the infected people we found only at the very beginning of the pandemic. In the German hot-spot Bavaria we found the results for the period from February 24 to April 20, 2020 the $\beta=0.22658$ with the non-linear regression. With the log-linear approach we found the quite similar value $\beta=0.23$.

At the end we can state that the estimation of the parameter $\beta$ is complicated, but successful in most of the considered countries and regions. The results of the solution of the minimum-problem (6) to evaluate $\beta$ are in most of the cases with respect to the fitting of the real data better than the results of the minimization of functional (5).

**Fig. 5:**
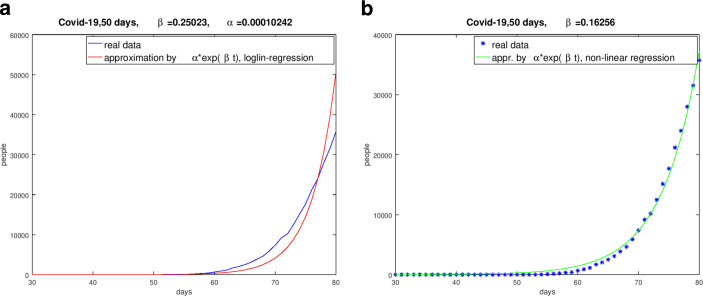
Comparison of the real data and the curves with the evaluated parameter $\beta$ for Italy from January 31, 2020 to March 20, 2020. **(a)** Result of Italy with the $\beta$-value from the logarithmic-linear regression, **(b)** Result of Italy with the $\beta$-value form the non-linear minimization

To illustrate the different quality and quantity of the $\beta$-estimation we use Italy as an example with Fig. 5. This was also confirmed by the comparison of the numerical simulations based on the evaluated $\beta$-values with the real data [15].

The interested readers are invited to make their own experiences with the real data of the beginning of the pandemic. The German data of the development of infected people from February 13, 2020 to of March 19, 2020 [14] are given in an Appendix and interested people can try to determine the parameter $\beta$ by themselves with their own programm codes (python, MATLAB,..) as an exercise. This can be done for other countries based on the data of the Johns-Hopkins University [17] also.

**Fig. 6:**
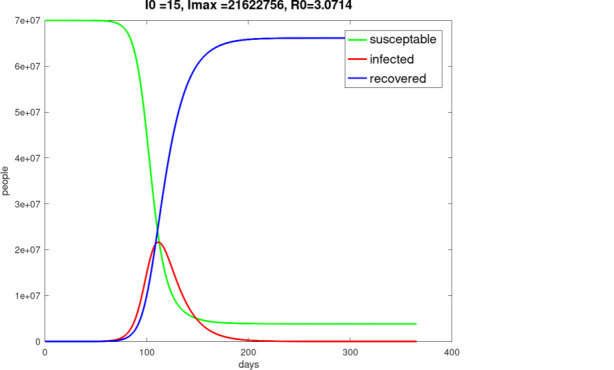
“Undisturbed” one-year pandemic course of Germany with initially 15 infected persons on day 0, starting on February 13, 2020

For the numerical solution of the *SIR* model initial value problem (1)–(3) we use a fourth-order Runge–Kutta method. The result with the $\beta$-value 0.215 for Germany is shown in Fig. 6. The term “undisturbed” means a propagation of the pandemic without any pharmaceutical or non-pharmaceutical measures by political and medical authorities.

As a time-step we used $\Delta\,t=1$ day. The initial values are $S(0)=N-15$, $I(0)=15$ and $R(0)=0$. As a population we assumed $N=70$ million^4^.

## Some properties of the *SIR* model

In the following we prove some typical properties of the *SIR* model. All these features can be transmitted to generalizations of *SIR*-type models, but owing to readability we will show it for the basic *SIR* model. A good survey on these issues are discussed in [41].

Because of the continuity of the right-hand side of the equation system (1)–(3) there is at least one local solution $(S,I,R):[t_{0},T]\to\mathbb{R}^{3}$ for given initial values $(S(t_{0}),I(t_{0}),R(t_{0}))=(S_{0},I_{0},R_{0})$ as a consequence of the existence theorem of Peano^5^.

### Theorem 1

For initial values $S_{0}> 0$, $I_{0}> 0$ and $R_{0}=0$ and positive parameters $\beta$ and $\gamma$ the solution $(S(t),I(t),R(t))$ has the properties

- $$I(t)> 0\quad\forall t\in[t_{0},\infty[$$,
- $$S(t)> 0$$ and $$\frac{dS}{dt}(t)<0\quad\forall t\in[t_{0},\infty[$$ and
- $$R(t)> 0$$ and $$\frac{dR}{dt}(t)> 0\quad\forall t\in]t_{0},\infty[$$.

### *Proof *

a) For the Eq. (2) we find

$$\frac{dI}{dt}=\left(\beta\frac{S}{N}-\gamma\right)I \Longrightarrow \frac{dI}{I}=\left(\beta\frac{S}{N}-\gamma\right)dt$$

$$\ln|I|=-\gamma t+\beta\int_{t_{0}}^{t}\frac{S}{N}\,dt+c$$

$$I(t)=I_{0}\exp\left[-\gamma t+\beta\int_{t_{0}}^{t}\frac{S}{N}\,dt\right]> 0$$

with $\exp(c)=I_{0}$. Using the initial value of $I$ one gets rid of the absolute value of $I$. b) The integration of Eq. (1) gives

$$\frac{dS}{dt}=-\beta\frac{I}{N}S\Longrightarrow S(t)=S_{0}\exp\left[\int_{t_{0}}^{t}\beta\frac{I}{N}\,dt\right]> 0\;.$$

Because of $\beta,\,S,\,I> 0$ follows $\frac{dS}{dt}<0$.c) The proof of property c) is analogous with those of b).∎

### Theorem 2

For the limits applies

$$\lim_{t\to\infty}S(t)=S_{\infty}> 0,\quad\lim_{t\to\infty}I(t)=I_{\infty}=0,\quad\lim_{t\to\infty}R(t)=R_{\infty}=N-S_{\infty}\;.$$

### *Proof *

It is easy to see that $S+I+R=N=\text{const.}$ and because of $S,I,R> 0$ and the monotonicity the existence of the limits follows. $S> 0$ and $\frac{dS}{dt}<0$ implies the existence of $S_{\infty}\in[0,N[$.

Case $S_{\infty}> 0$: The assumption $I_{\infty}> 0$ and therefore $\lim_{t\to\infty}\frac{dS}{dt}(t)=\text{const.}<0$ contradicts the result $S> 0$.

Case $S_{\infty}=0$: We assume $I_{\infty}> 0$. Then for large $t$ the term $\frac{\beta S}{N}-\gamma$ is strictly negative and the contradiction follows from Eq. (2) for $I$.

This implies $I_{\infty}=0$. For the third limit follows $R_{\infty}=N-S_{\infty}-I_{\infty}= N-S_{\infty}$.∎

In the general discussion of a pandemic the term “herd immunity” plays an important role. The mathematical background should be mentioned in the following. Considering the differential equation for the infected people

$$\frac{dI}{dt}=I\left(\beta\frac{S}{N}-\gamma\right)$$

it follows

$$\frac{dI}{dt}> 0\Longleftrightarrow S> \frac{\gamma}{\beta}N\quad \text{and}\quad\frac{dI}{dt}<0\Longleftrightarrow S<\frac{\gamma}{\beta}N\;.$$

Consequently we experience an abatement of the pandemic, if we have

$$S=N-I-R<\frac{\gamma}{\beta}N\Longleftrightarrow I+R> N-\frac{\gamma}{\beta}N:=H\;.$$

For the German realistic values $\beta\approx 0.21$ and $\gamma\approx 0.07$ we got with

$$H=\frac{2}{3}N$$

the term “herd-immunity”. We summarize this discussion in the following theorem.

### Theorem 3

The pandemic ends, if with

$$I+R> H$$

the herd immunity is reached.

It must be said that models which include the compartment of vaccinated people $V$ influences the herd immunity positively because the herd immunity is reached if $I+R+V$ is greater than $H$.

In the daily reports of the state medical-hygienic institutions (the Robert-Koch Institut (RKI) is responsible for that in Germany) by this they use the term “reproduction number” ${\cal R}$ which is defined as

$${\cal R}=\frac{\beta}{\gamma}\frac{S}{N}\;.$$

At the beginning of the pandemic we have $S\approx N$ and the reproduction number simplifies to

$${\cal R}_{0}=\frac{\beta}{\gamma}\;,$$

the basic reproduction number. ${\cal R}_{0}$ of Germany in January 2020 was approximately equal to 3. Considering the Eq. (2) it is obvious that there is no pandemic, i.e. the development of $I$ will decrease, if we have the situation ${\cal R}_{0}<1$. At the beginning of the consideration we have $S\approx N$ and from (2) follows

$$\frac{dI}{dt}\approx(\beta-\gamma)I$$

with the solution

$$I(t)=I(0)\exp(\beta-\gamma)=I(0)\exp(\gamma({\cal R}_{0}-1))\;.$$

We can also conclude that the number of infected people will decrease if ${\cal R}<1$ applies. Will we summarize this behavior in the following theorem.

### Theorem 4

The pandemic does not start if ${\cal R}_{0}<1$ applies.The number of infected people decreases if ${\cal R}<1$ applies.

If we combine the differential equations (1) and (2) by a formal division we get with $\rho=\frac{\gamma}{\beta}N$

7 $$\frac{dI}{dS}=\frac{I(\beta S-\gamma N)}{I\beta S}=-1+\frac{\rho}{S}\;.$$

The integration of (7) gives with

8 $$E=I(t)+S(t)-\rho\log S(t)=I(0)+S(0)-\rho\log S(0)=\text{const.}\;\;$$

a conserved quantity (also called first integral), which is constant on the trajectories of the $(I,S)$-phase space. The Eq. (8) gives with

$$I_{\text{max}}=-\rho+\rho\log\rho+I(0)+S(0)-\rho\log S(0)\approx N-\rho+\rho\log\left(\frac{\rho}{S(0)}\right)$$

the maximal number of infected people during the pandemic for $S(t)=\rho$, because the function $f(S)=\rho\log S-S$ has its maximum at $S=\rho$. At the end we get with $S(0)=N$ and $I(0)=0$ (and $\rho=\frac{N}{{\cal R}_{0}}$)

$$I_{\text{max}}=\frac{N}{{\cal R}_{0}}({\cal R}_{0}-1-\log{\cal R}_{0})\quad(\approx 21\;\text{million}).$$

## Analytic solution of the *SIR* equation system

The general *SIR* initial value problem will be solved numerically, especially if the model parameters are not constant. For example it’s possible to work with a time-dependent function $\beta$ instead of a constant value.

But for the investigation of the long-time behavior of the pandemic and for certain stages of the pandemic analytical solutions are possible (see for example [25]).

The formal division of the equations (1) and (3) gives

$$\frac{dS}{dR}=-\frac{S\beta}{N\gamma}=-\frac{S}{\rho}\;,$$

with the solution

$$S(t)=S_{0}\exp\left[-\frac{R}{\rho}\right]\;.$$

Therefore Eq. (3) implies

9 $$\frac{dR}{dt}=\gamma I=\gamma(N-R-S)=\gamma\left(N-R-S_{0}\exp\left[-\frac{R}{\rho}\right]\right)\;.$$

For small $\frac{R}{\rho}$ it is possible to approach the exponential function by the Taylor expansion around $\frac{R}{\rho}$

$$\exp\left[-\frac{R}{\rho}\right]\approx 1-\frac{R}{\rho}+\frac{1}{2}\frac{R^{2}}{\rho^{2}}+\dots$$

and the Eq. (9) can be approximated by the Riccati equation (see for example [38])

10 $$\frac{dR}{dt}=\gamma\left(N-S_{0}+\left(\frac{S_{0}}{\rho}-1\right)R-\frac{S_{0}R^{2}}{2\rho^{2}}\right)$$

with the solution

11 $$R(t)=\frac{\rho^{2}}{S_{0}}\left(\left(\frac{S_{0}}{\rho}-1\right)+\alpha\tanh\left(\frac{\alpha\gamma t}{2}-\phi\right)\right)$$

using $\alpha$ and the phase $\phi$, defined by

$$\alpha=\sqrt{(\frac{S_{0}}{\rho}-1)^{2}+2\frac{S_{0}(N-S_{0})}{\rho^{2}}}\quad\text{and}\quad\phi=\tanh^{-1}\left(\frac{1}{\alpha}\left(\frac{S_{0}}{\rho}-1\right)\right)\;.$$

With the Eq. (3) and the formula (11) we get for the infected people the approximation^6^

12 $$I(t)=\frac{1}{\gamma}\frac{dR}{dt}=\frac{\alpha^{2}\rho^{2}}{2S_{0}}\text{sech}^{2}\left(\frac{\alpha\gamma t}{2}-\phi\right)\;.$$

Using this approximation Kermack/McKendrick [26] could approach the regime of the plague epidemic of 1905/1906 in Bombay/India very well. The interested reader is invited to discuss this formula as an exercise (see also the Appendix).

If we suppose $S_{0}> \rho$ and $I_{0}=0$ it applies $\alpha=\frac{S_{0}}{\rho}-1$ and therefore

$$R(t)=\frac{\rho^{2}}{S_{0}}\left(\frac{S_{0}}{\rho}-1\right)\left(1+\tanh\left(\frac{\gamma}{2}(\frac{S_{0}}{\rho}-1)t-\phi\right)\right)\;.$$

And with $\lim_{t\to\infty}\tanh\left(\frac{\alpha\gamma t}{2}-\phi\right)=1$ we get an approximation for the magnitude of the pandemic $R(\infty)=N-S(\infty)$

$$R(\infty)=2\rho\left(1-\frac{\rho}{S_{0}}\right)\;.$$

If we have $S_{0}=\rho+\epsilon$, $\epsilon> 0$, at the beginning of the pandemic we get

$$R(\infty)=2\rho\frac{\epsilon}{\rho+\epsilon}=2\epsilon\;,$$

and $S(\infty)=N-2\epsilon$. This means during the pandemic the number of susceptible people was reduced by $2\epsilon$.

Up to now we neglect the demographic dynamics in the *SIR* model. This is possible if we consider only short time periods of a pandemic. If we are interested in longer time periods we have to respect the birth and death rates in the model. With a birth rate $g$ and a death rate $d$ the *SIR* model is changed to

13 $$\begin{aligned}\frac{dS}{dt} & = & gN-\beta\frac{S}{N}I-dS\end{aligned}$$

14 $$\begin{aligned}\frac{dI}{dt} & = & \beta\frac{S}{N}I-\gamma I-dI\end{aligned}$$

15 $$\begin{aligned}\frac{dR}{dt} & = & \gamma I-dR\;.\end{aligned}$$

In contrast to the basic *SIR* model the quantity $N$ is not constant in general. The addition of the equations (13)–(15) gives the equation of the population dynamics

$$\frac{dN}{dt}=(g-d)N\;.$$

## Extensions of the *SIR* model

In the following some extensions of the *SIR* model will be treated and adumbrated. The general assumptions are not changed. We assume a homogeneous distribution of people in a certain region (state, area). The extension consists in a further subdivision of the compartments of the *SIR* model [22]. On the other hand stochastic phenomenona will be introduced. The numerical solutions for the ode systems carried out were done with a fourth-order Runge–Kutta method.

### *SEIR* model

As we mentioned above there are generalizations of the *SIR* model. Because of the latency between the infection of people with the virus and the capability to transmit the virus one can divide the compartment of infected people into a group of infected people who are not infectious, called the group of exposed people $E$, and the group of infected and infectious people $I$. This reflection leads to the *SEIR* model with the differential equation system

16 $$\begin{aligned}\frac{dS}{dt} & = & -\beta\frac{S}{N}I\end{aligned}$$

17 $$\begin{aligned}\frac{dE}{dt} & = & \beta\frac{S}{N}I-\alpha E\end{aligned}$$

18 $$\begin{aligned}\frac{dI}{dt} & = & \alpha E-\gamma I\end{aligned}$$

19 $$\begin{aligned}\frac{dR}{dt} & = & \gamma I\;.\end{aligned}$$

The new parameter $\alpha$ stands for the transmission rate of exposed persons to infectious ones. The latency period is the reciprocal of $\alpha$.

**Fig. 7:**
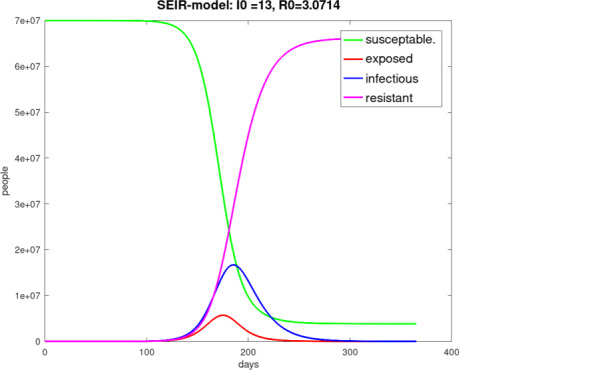
“Undisturbed” one-year pandemic course of Germany with initially 15 infected persons on day 0, starting on February 13, 2020, $\beta=0.215$, $\gamma=0.07$, $\alpha=0.25$, $N=70$ million, $I(0)=15$, $S(0)=N-I(0)$, $E(0)=R(0)=0$

The properties of this model are quite similar to those of the *SIR* model, it means for example the positivity, the boundedness and the behavior of monotony. In Fig. 7 a typical result of the application of the *SEIR* model is shown.

### *SIR*-$X$ model

At the beginning of the COVID-19 pandemic was found a super-linear and sub-exponential growth of the number of infected people, for example in China and Austria. The phenomenon could not really be well described with the *SIR* or *SEIR* model. A better description was possible with the *SIR*-$X$ model [32], where the compartment $I$ of infected people was subdivided into symptomatic and quarantined people – compartment $X$ and other infected people $I$. This can be described by the following equation system.

20 $$\begin{aligned}\frac{dS}{dt} & = & -\beta\frac{S}{N}I-\eta_{0}S\end{aligned}$$

21 $$\begin{aligned}\frac{dI}{dt} & = & \beta\frac{S}{N}I-\gamma I-\eta_{0}I-\eta I\end{aligned}$$

22 $$\begin{aligned}\frac{dR}{dt} & = & \gamma I+\eta_{0}S\end{aligned}$$

23 $$\begin{aligned}\frac{dX}{dt} & = & (\eta+\eta_{0})I\;.\end{aligned}$$

**Fig. 8:**
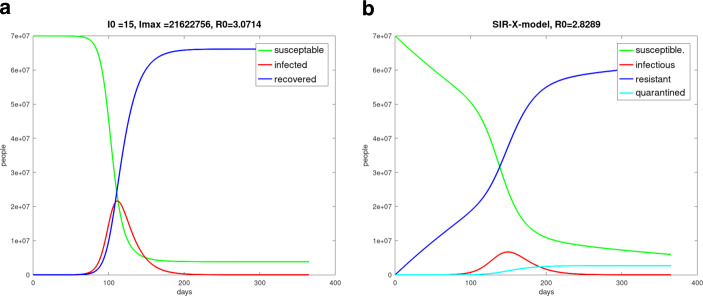
**(a)** *SIR* model simulation, one year pandemic course of Germany with initially 15 infected persons on day 0 from February 13, 2020 to February 13, 2021, $\beta=0.215$, $\gamma=0.07$, $\alpha=0.25$, $N=70$ million, $I(0)=15$, $S(0)=N-I(0)$, $R(0)=0$; **(b)** *SIR*-$X$ model simulation, one year pandemic course of Germany with initially 15 infected persons on day 0 from February 13, 2020 to February 13, 2021, $\beta=0.215$, $\gamma=0.07$, $\alpha=0.25$, $N=70$ million, $I(0)=15$, $S(0)=N-I(0)$, $R(0)=0$, $\eta_{0}=\eta=0.003$

The parameter $\eta_{0}$ describes measures to protect vulnerable people, and $\eta$ is responsible for the quarantine efforts. With the Fig. 8 the *SIR* simulation was compared to the *SIR*-$X$ simulation. For the numerical solution we used a fourth-order Runge–Kutta method.

### A multi-compartment model

Contreras et al. [19] considered the following compartments in a huge extension of the *SIR* model. This model arises in the actual research of physicists and mathematicians dealing with the modeling of the COVID-19 pandemic.

- $$S$$ – susceptible people
- $E^{Q}$ – exposed, non-infectious quarantined people
- $E^{H}$ – unrecognized infected, non-infectious people
- $I^{Q}$ – infected and infectious quarantined people
- $I^{H}$ – unrecognized infected and infectious people
- $I^{H,s}$ – unrecognized infected and infectious people with typical symptoms
- $R$ – removed people (restored to health or dead).

Unrecognized infected and infectious people without typical symptoms $I^{H,a}$ follow from

$$I^{H,a}=I^{H}-I^{H,s}\;.$$

For

$$x=[S,E^{Q},E^{H},I^{Q},I^{H},I^{H,s},R]^{T}$$

a dynamical system

$$\dot{x}=F(x)\;,\quad F:\mathbb{R}^{6}\to\mathbb{R}^{6}$$

was formulated. As examples the equations for $I^{Q}$ and $I^{H}$ shall be given.

$$\frac{dI^{Q}}{dt} = \underbrace{\rho E^{Q}-\gamma I^{Q}+N^{\text{test}}}_{\text{propagation dynamics}}+\underbrace{(\chi_{s,r}(1-\xi)+\chi_{r}\xi)N^{\text{traced}}}_{\text{tracing of contacts}}$$

$$\frac{dI^{H}}{dt} = \underbrace{\rho E^{H}-\gamma I^{H}-N^{\text{test}}}_{\text{propagation dynamics}}-\underbrace{(\chi_{s,r}(1-\xi)+\chi_{r}\xi)N^{\text{traced}}}_{\text{tracing of contacts}}+\underbrace{\frac{S}{M}\Psi_{t}}_{\text{external influences}}$$

A problem of such multi-compartment models consists in the finding of appropriate parameters. The number of traced and tested people are known. But the parameters $\rho$, $\chi_{s,r}$, $\chi_{r}$ for example must be fitted based on real observed data.

On the other hand, it must be respected that the parameters are in general time-dependent functions. Only short time periods can be simulated with constant parameters. But this works and yields valuable results of the influences of the contact parameters on the development of the pandemic. This is well documented in [19].

### A stochastic framework

Random influences cannot be fitted by the standard models [2–5]. But there are some uncertainties which cannot be embraced by the model parameters. And this is the reason to extend the deterministic models to stochastic ones.

Stochastic pioneers like Norbert Wiener and Kiyoshi Itó (see [23]) introduced the mathematical basics of stochastic analysis and stochastic processes. Key concepts like Brownian motion (Wiener process)^7^ or the Itó-integral innovated the theory of stochastic differential equations. This theory was extended in present time by B. Liu [30].

Because of the fact that a Wiener process $W_{t}$ is not differentiable, an equation like

$$\frac{dX_{t}}{dt}=F(X_{t})+G(X_{t})\frac{dW_{t}}{dt}$$

do not stack up because of the missing of differentiability of $W_{t}$, where $X_{t}$ is a random variable and $W_{t}$ is a Wiener process. That is the reason for the integral formulation

24 $$dX_{t}=F(X_{t})dt+G(X_{t})dW_{t}\;.$$

Eqs. (24) are called “stochastic differential equations” (sde, see for example [37]). A formal solution of (24) for a given initial state $X_{0}$ is of the form

25 $$X_{t}=X_{0}+\int_{0}^{t}F(X_{t})dt+\int_{0}^{t}G(X_{t})dW_{t}\;.$$

The first integral of (25) is a classic Riemann-integral, but the second integral

26 $$\int_{0}^{t}G(X_{t})dW_{t}$$

is not covered by the classic integration because of the mad properties of the Wiener process $W_{t}$. Itó introduced for integrals like (26) the concept of the “Itó integral” and thus he showed a way to solve stochastic differential equations with the formula (25). The stochastic process $X_{t}$ from Eq. (25) is called an **Itó process**.

Now we can augment the deterministic model equation system to the stochastic differential equation system

27 $$\begin{aligned}dS_{t} & = & -\beta\frac{S_{t}}{N}I_{t}\,dt+\sum_{j=1}^{k}g_{1j}(S_{t},I_{t})dW_{jt}\end{aligned}$$

28 $$\begin{aligned}dI_{t} & = & \left(\beta\frac{S_{t}}{N}I_{t}-\gamma I_{t}\right)\,dt+\sum_{j=1}^{k}g_{2j}(S_{t},I_{t})dW_{jt}\;.\end{aligned}$$

The index $t$ does not mean a time derivative. $I_{t}$ and $S_{t}$ denote stochastic processes and $W_{t}=(W_{1t},\dots,W_{kt})^{T}$ is a vector of independent Wiener processes with the main characteristic $W_{jt}-W_{js}\sim N(0,\sqrt{t-s})$, $t> s$ and the independence of $W_{kt}$ and $W_{ks}$ for $t\neq s$ ($k=1,2$). Since $N=S_{t}+I_{t}+R_{t}=\text{const.}$, we have $R_{t}=N-S_{t}-I_{t}$, and since the equations (27) and (28) do not depend on $R_{t}$, it is not necessary to consider an equation for $R_{t}$.

To get an idea of the matrix $G=(g_{ij})_{i=1,2,j=1,\dots,k}$ we follow [3] to consider with

$$\Delta X_{t}=(\Delta S_{t},\Delta I_{t})^{T}=(\Delta X_{1t},\Delta X_{2t})^{T}$$

the change of the random variables $S_{t}$ and $I_{t}$ at time $t$. We divide the interval $[0,t[$ into small sub-intervals of length $\Delta t$. The interval of length $\Delta t$ was divided further into smaller sub-intervals of the length $\Delta t_{j}=t_{j}-t_{j-1},\,j=1,\dots,n$ with $t_{0}=t,\,t_{n}=t+\Delta t$ and $\sum_{j=1}^{n}\Delta t_{j}=\Delta t$. For the changes $\Delta X_{t}$ it is

$$\Delta X_{t}=\sum_{j=1}^{n}\Delta X_{t_{j}}\;.$$

For sufficiently small steps $\Delta t_{j}$ one can assume that the randow variables $\{\Delta X_{t_{j}}\}$ on the interval $\Delta t$ are independent and identically distributed. For $n$ sufficiently large the Central Limit Theorem implies that $\Delta X$ has an approximate normal distribution with mean $E(\Delta X_{t})$ and covariance matrix $\text{COV}(\Delta X_{t})$ e.g.

$$\Delta X_{t}-{E}(\Delta X_{t})\approx N({\bf 0},\text{COV}(\Delta X_{t}))\;.$$

The expectation of $\Delta X$ to order $\Delta t$ is the change that occurs $(+1\;\text{or}\;-1)$ times the probability^8^

$${E}(\Delta X)=\left(\begin{array}[]{c}-\beta SI/N\\ \beta SI/N-\gamma I\end{array}\right)\Delta t=:F\Delta t$$

and the covariance matrix of $\Delta X$ to order $\Delta t$

$$\text{COV}(\Delta X) = {E}(\Delta X(\Delta X)^{T})={E}\left(\begin{array}[]{cc}(\Delta S)^{2}&\Delta S\Delta I\\ \Delta S\Delta I&(\Delta I)^{2}\end{array}\right)$$

$$= \sum_{j=1}^{2}p_{j}\Delta X_{j}(\Delta X_{j})^{T}=\left(\begin{array}[]{cc}\beta SI/N&-\beta SI/N\\ -\beta SI/N&\beta SI/N+\gamma I\end{array}\right)\Delta t=:C\Delta t$$

To write the SDE for the *SIR* stochastic process either the square root of the covariance matrix $C\Delta t$ is required, or alternatively, a matrix $G$ so that $GG^{T}=C$. The following matrix $G$ has this latter property ($G$ is not unique)^9^. The matrix $G$ is straightforward to compute as each component represents the square roots of the rates as given in Table 1.

**Table 1:** *SIR* assumptions

Event	Change $(\Delta S,\Delta I)$	Rate	Probability
Infected	$(-1,+1)$	$r_{1}=\beta IS/N$	$p_{1}=r_{1}\Delta t$
Removed	$(0,-1)$	$r_{2}=\gamma I$	$p_{2}=r_{2}\Delta t$

$$G=\left(\begin{array}[]{cc}-\sqrt{\beta SI/N}&0\\ \sqrt{\beta SI/N}&-\sqrt{\gamma I}\end{array}\right)$$

Then we have $\Delta X_{t}=F(X_{t})\Delta t+G(X_{t})dW_{t}$, with

$$F(X_{t})=\left(\begin{array}[]{c}-\beta\frac{IS}{N}\\ \beta\frac{SI}{N}-\gamma I\end{array}\right)\;,$$

$\Delta W_{t}=(\Delta W_{1t},\Delta W_{2t})^{T}$ and $\Delta W_{1t}$, $\Delta W_{2t}\sim N(0,\Delta t)$. The limit $\Delta t\to 0$ gives with

$$dX_{t}=F(X_{t})dt+G(X_{t})dW_{t}$$

the relevant SDE system. Together with initial data $S_{0}$ and $I_{0}$ a stochastic *SIR* model is defined.

Let us now discuss the *SEIR* model (16)–(19). Analogically with the *SIR* model we consider the vector of stochastic processes $X_{t}=(S_{t},E_{t},I_{t})^{T}$ and the vector of independent Wiener processes $W_{t}=(W_{1t},W_{2t},W_{t3})^{T}$. The stochastic *SEIR* model is of the form

29 $$dX_{t}=F(X_{t})dt+G(X_{t})dW_{t}\;.$$

The fourth equation for $R$ can be considered separately. For the determination of the diffusion matrix $G$ we look at the changing rates in Table 2.

**Table 2:** *SEIR* assumptions

Event	Change $(\Delta S,\Delta E,\Delta I)$	Rate	Probability
Infected	$(-1,+1,0)$	$r_{1}=\beta IS/N$	$p_{1}=r_{1}\Delta t$
Infectious	$(0,-1,+1)$	$r_{2}=\alpha E$	$p_{2}=r_{2}\Delta t$
Removed	$(0,0,-1)$	$r_{3}=\gamma I$	$p_{3}=r_{3}\Delta t$

Analogically to proceeding with the *SIR* model we find with

$$\text{COV}(\Delta X)={E}(\Delta X(\Delta X)^{T})={E}\left(\begin{array}[]{ccc}(\Delta S)^{2}&\Delta S\Delta E&\Delta S\Delta I\\ \Delta S\Delta E&(\Delta E)^{2}&\Delta E\Delta I\\ \Delta S\Delta I&\Delta I\Delta E&(\Delta I)^{2}\end{array}\right)$$

$$=\sum_{j=1}^{3}p_{j}\Delta X_{j}(\Delta X_{j})^{T}=\left(\begin{array}[]{ccc}\beta SI/N&-\beta SI/N&0\\ -\beta SI/N&\beta SI/N+\alpha E&-\alpha E\\ 0&-\alpha E&\alpha E+\gamma I\end{array}\right)\Delta t=:C\Delta t$$

the covariance matrix of $\Delta X=(\Delta S,\Delta E,\Delta I)^{T}$ to order $\Delta t$. The finding of a matrix $G$ with $GG^{T}=C$ is straightforward with the result

$$G=\left(\begin{array}[]{ccc}-\sqrt{\beta SI/N}&0&0\\ \sqrt{\beta SI/N}&-\sqrt{\alpha E}&0\\ 0&\sqrt{\alpha E}&-\sqrt{\gamma I}\end{array}\right)\;.$$

Then we have $\Delta X_{t}=F(X_{t})\Delta t+G(X_{t})dW_{t}$, with

$$F(X_{t})=\left(\begin{array}[]{c}-\beta\frac{IS}{N}\\ \beta\frac{SI}{N}-\alpha E\\ \alpha E-\gamma I\end{array}\right)\;,$$

$\Delta W_{t}=(\Delta W_{1t},\Delta W_{2t},\Delta W_{3t})^{T}$ and $\Delta W_{jt}\sim N(0,\Delta t),\,j=1,2,3$. The limit $\Delta t\to 0$ gives with

$$dX_{t}=F(X_{t})dt+G(X_{t})dW_{t}$$

the relevant SDE system.

**Fig. 9:**
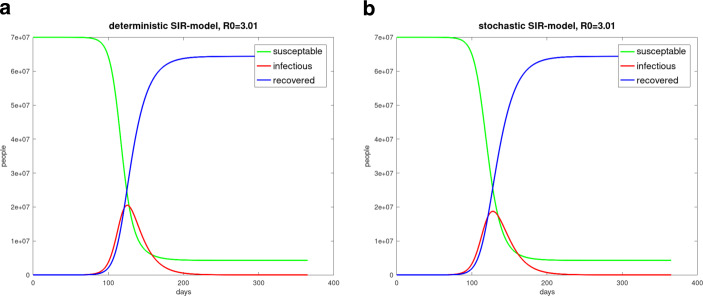
**(a)** Deterministic simulation of the one-year pandemic regime in Germany; **(b)** Stochastic *SIR* simulation of the one-year pandemic regime in Germany. $\beta=0.215$, $\gamma=0.07$, $N=70$ million, $I(0)=15$, $S(0)=N-I(0)$, $R(0)=0$

The sde-system (27)–(28) was solved with the Euler–Maruyama-method which was explained for example in [36] or [11]. The results of the simulations are pictured in Fig. 9.

The comparison of deterministic and stochastic simulations showed that the results of the deterministic simulations overestimate the height of the infected curve. The stochastic simulations results in a decreased maximum of infected people. The maximum value of infected people of the deterministic simulation was 20 505 724 compared to the stochastic one of 18 721 628. The time-step $h=0.5$ day was used. The stochastic result was the mean of 50 simulated paths.

## Non-pharmaceutical interventions to prevent the pandemic

In all countries concerned by the COVID-19 pandemic, lockdown measures of social life have been discussed. In Germany, a first lockdown started on March 16, 2020. The effects of social distancing to decrease the infection rate can be modeled by a modification of the *SIR* model. Now, we consider $\beta$ in the equation system (1)–(3) as a time-dependent function (instead of $\beta=\beta_{0}=\text{const.}$ in the original *SIR* model). The limitations of contacts to 20% of normality^10^ starting at time $t_{0}$ can be described for example by the function

$$\beta(t)=\left\{\begin{array}[]{ll}0.2\beta_{0}&\text{for }t_{0}\leq t\leq t_{1}\\ \beta_{0}&\text{for }t> t_{1},\;t<t_{0}\end{array}\right.$$

**Fig. 10:**
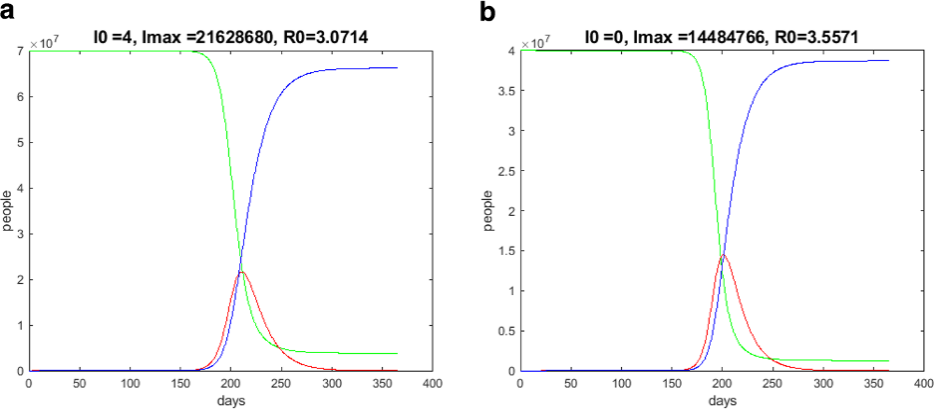
Results with lockdowns; $S$—green, $I$—red, $R$—blue; 30 days lockdown, starting on March 16, 2020. **(a)** German progression over one year, starting at the end of January 2020; **(b)** Spanish progression over one year, starting at the end of January 2020

If we respect the chosen starting day of the German lockdown, March 16, 2020 (this conforms to the 46th day of the year concerned starting at the end of January 2020), and we work with

$$\beta(t)=\left\{\begin{array}[]{ll}\beta_{0}&\text{for }t<46\\ 0.2\beta_{0}&\text{for }46\leq t\leq 76\\ \beta_{0}&\text{for }t> 76,\;t<46,\end{array}\right.$$

then we get the result pictured in Fig. 10a.

The numerical tests showed that a very early start of the lockdown, resulting in a reduction of the infection rate $\beta_{0}$, causes the typical Gaussian curve to be delayed by $I$; however, the amplitude (maximum value of $I$) does not really change.

It is known from other pandemics, such as the Spanish flu [9, 33] or the swine flu, that the development of the number of infected people looks like a Gaussian curve. The interesting points in time are those where the acceleration of the numbers of infected people increases or decreases, respectively.

These are the points in time where the curve of $I$ changes from a convex to a concave behavior or vice versa. The convexity or concavity can be controlled by the second derivative of $I(t)$.

Let us consider Eq. (2) and suppose that $\beta$ is constant. By differentiation of (2) and the use of (1), I get

$$\frac{d^{2}I}{dt^{2}} = \frac{\beta}{N}\frac{dS}{dt}I+\frac{\beta}{N}S\frac{dI}{dt}-\gamma\frac{dI}{dt}$$

$$= -\frac{\beta}{N}^{2}SI^{2}+\left(\frac{\beta S}{N}-\gamma\right)\left(\frac{\beta S}{N}-\gamma\right)I$$

$$= \left[\left(\frac{\beta S}{N}-\gamma\right)^{2}-\left(\frac{\beta}{N}\right)^{2}SI\right]I\;.$$

With that, the $I$-curve will change from convex to concave we have

30 $$\left(\frac{\beta S}{N}-\gamma\right)^{2}-\left(\frac{\beta}{N}\right)^{2}SI=0\Longleftrightarrow I=\frac{\left(\frac{\beta S}{N}-\gamma\right)^{2}N^{2}}{\beta^{2}S}\;.$$

The switching time follows

31 $$t_{0}=\sup_{t}\left\{t> 0,\,I(t)> \left(\left(\frac{\beta S(t)}{N}-\gamma\right)^{2}N^{2}\right)\Big/\left(\beta^{2}S(t)\right)\right\}\;.$$

A lockdown starting at $t_{0}$ (assigning $\beta=\kappa\beta_{0}$,  $\kappa\in[0,1[$) up to a point in time $t_{1}=t_{0}+\Delta_{t}$, with $\Delta_{t}$ as the duration of the lockdown in days, will be denoted as a dynamical lockdown (for $t> t_{1}$, $\beta$ is reset to the original value $\beta_{0}$).

**Fig. 11:**
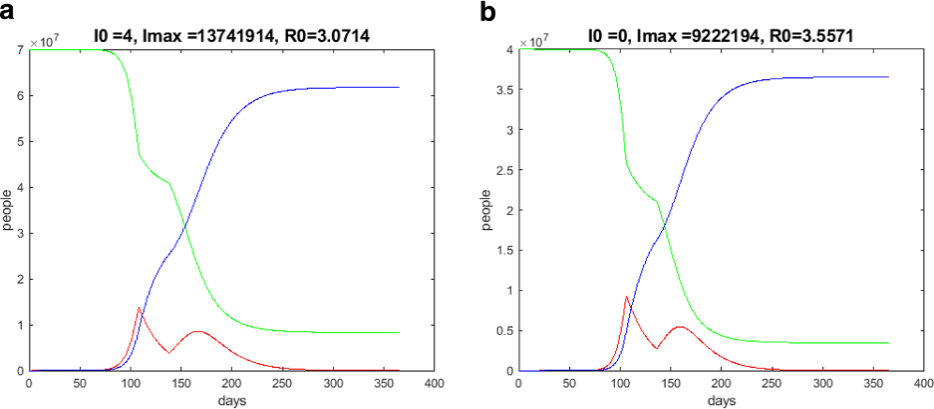
Results over one year; $S$—green, $I$—red, $R$—blue. **(a)** German progression over one year, starting at the end of January 2020, dynamical lockdown; **(b)** Spanish progression over one year, starting at the end of March 2020, dynamical lockdown

**Fig. 12:**
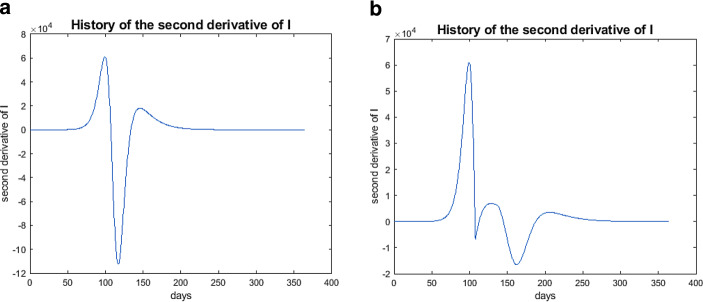
Typical history of the second derivatives of $I$. **(a)** History of the second derivative of $I$; **(b)** History of the second derivative of $I$ with dynamical lockdown

$t_{0}$ indicates the point in time up to which the growth rate increases and after which it decreases. Fig. 11a shows the result of such a computation of a dynamical 30-day lockdown. I obtained $t_{0}=108$ ($\beta=0.2\beta_{0}$). The result is significant. In Fig. 12a, a typical behavior of $\frac{d^{2}I}{dt^{2}}$ is plotted (in Fig. 12b, $\frac{d^{2}I}{dt^{2}}$ in the dynamical lockdown case).

The result of a dynamical 30-day lockdown for Spain is shown in Fig. 11b, where I found $t_{0}=106$ ($\beta=0.2\beta_{0}$).

The lockdown-simulations show that one can win time with such measures, but a terminable social distancing moves the curve of infected people by the period of lockdown days into the future. The experiences of Germany and other countries concerned show that limited lockdown measures without any other relevant interventions do not solve the pandemic problems but flatten the curve of infected people for the lockdown period only.

What are “relevant” interventions? At the end of 2020 mankind was given the first efficacious COVID-19 vaccines, and the immunization campaign could be started. The influence of the COVID-19 vaccination will be discussed in the next section.

## Pharmaceutical interventions by vaccination

**Fig. 13:**
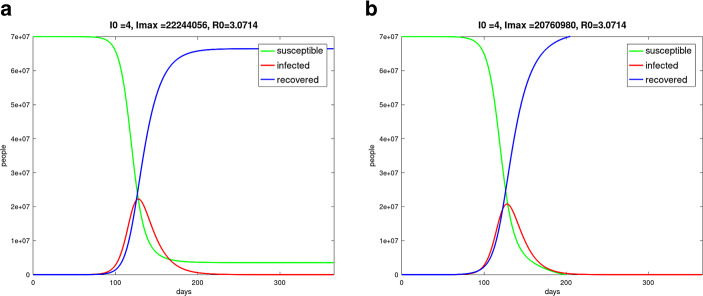
**(a)** Result without vaccination; **(b)** Result with the vaccination function $V(t)$

**Fig. 14:**
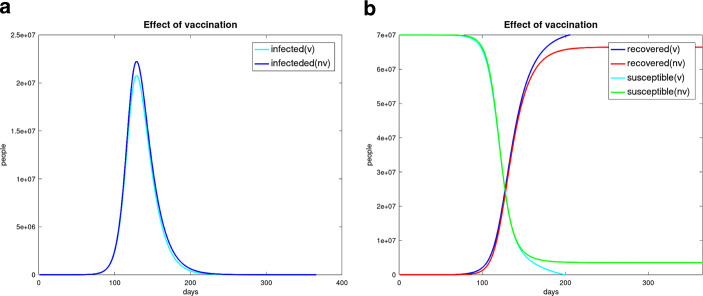
Effect of vaccination Infected people (**a**); Susceptible and removed people (**b**)

The vaccination campaign should be factored in the basic *SIR* model. Every vaccination reduces the compartment of susceptible people and increases the removed group. This leads to the modified *SIR*-equation system

32 $$\begin{aligned}\frac{dS}{dt} & = & -\beta\frac{S}{N}I-V\end{aligned}$$

33 $$\begin{aligned}\frac{dI}{dt} & = & \beta\frac{S}{N}I-\gamma I-\lambda V\end{aligned}$$

34 $$\begin{aligned}\frac{dR}{dt} & = & \gamma I+V\;,\end{aligned}$$

where $V$ is the amount of vaccinated people per day, which is a prescribed time-dependent function. If we use a vaccination function

$$V(t)=\left\{\begin{array}[]{ll}0&\text{\leavevmode\nobreak\ for\leavevmode\nobreak\ }t<50\\ 50\,000\sin((t-50)\pi/100-\pi/2)&\text{\leavevmode\nobreak\ for\leavevmode\nobreak\ }50\leq t\leq 250\\ 0&\text{\leavevmode\nobreak\ for\leavevmode\nobreak\ }t> 250\end{array}\right.$$

(it means a vaccination of 10 million people), we get the result of an exemplary simulation pictured in the Figures 13 and 14.

## A mathematical diffusion model to respect spatial virus propagation

What is a good choice of quantity to describe the COVID-19 spread? The World Health Organization (WHO) and national health institutions measure the COVID-19 spread with the seven-day incidence (WHO also uses the Fourteen-days incidence) of people with COVID-19 per 100 000 inhabitants. In Germany, it is possible to control or trace the history of people with COVID-19 by local health institutions if the seven-day incidence has a value less than 50. However, between the end of December 2020 and the beginning of January 2021, the averaged incidence was about 140 and, in some hotspot federal states, such as Saxony, it was greater than 300. At the end of March 2020 and at the beginning of April 2020, the incidences changed dramatically. However, in general, one considers a different pandemic development from one federal state to another and this situation needs to be respected with the consideration of diffusion phenomena.

If the social and economical life should be sustained, there are several possibilities of transmitting the COVID-19 virus. Among others, the following ones to be mentioned are:

- commuters and employees on the way to their office or to their position of employment, especially medical and nursing staff;
- pupils and teachers in schools and on the way to school;
- people buying everyday necessities using shopping centers;
- postmen, suppliers and deliverers.

All of these activities take place during so-called lockdowns in Germany, with the result of an ongoing propagation of the pandemic. Furthermore, the unavailable center of power in the decentralized federal states of Germany often leads to solo efforts of some federal states.

From countries, such as China or Singapore, with quite different civilization and cultural traditions than those in Germany, it is known that the virus propagation could be stopped with very rigorous measures such as the strict prohibition of social and economic life. Those ones mentioned-above are absolutely forbidden.

This is inconceivable in countries like Germany, Austria, the Netherlands or other states with a western understanding of freedom and self-determination. However, as a consequence of such a western lifestyle, they have to live with more or less consecutive activity of the COVID-19 pandemic. This is the reason for the following trial: to describe one aspect of the pandemic by a diffusion model. In connection with the pandemic, diffusion has been discussed, e.g., in [1, 7, 12]. The diffusion being a central process in many biological, social, chemical and physical systems is considered in [6, 44]. A similar model but in another context has been discussed in [13].

Within the diffusion concept discussed here, the seven-day incidence, denoted by $s$, serves as the quantity that is influenced by its gradients between different levels of incidence in the federal states of Germany.

The mathematical model of diffusion of a certain quantity $c$ is given by [21]:

35 $$\frac{\partial c}{\partial t}=\nabla\cdot(D\nabla c)+q\quad\text{in}\;[t_{0},T]\times\Omega,$$

where $\Omega\subset\mathbb{R}^{2}$ is the region that will be investigated, here the national territory of Germany, $D$ is a diffusion coefficient, depending on the locality $x\in\Omega$, $[t_{0},T]$ is the time interval of interest, and $q$ is a term that describes sources or sinks.

Now the seven-day incidence $s$ should be considered as such a quantity with the term $q$ that describes the possibility of infections. In addition to Eq. (35), one needs to define initial conditions for $s$, such as, e.g.,

36 $$s(x,t_{0})=s_{0}(x),\quad x\in\Omega\,,$$

and boundary conditions,

37 $$\alpha s+\beta\nabla s\cdot\vec{n}=\gamma\quad\text{in}\;[t_{0},T]\times\partial\Omega\,,$$

where $\alpha,\beta$ and $\gamma$ are real coefficients, $\partial\Omega=:\Gamma$ denotes the boundary of the region $\Omega$, and $\nabla_{n}s=\nabla s\cdot\vec{n}$ is the directional derivative of $s$ in the direction of the outer normal vector $\vec{n}$ on $\Gamma$. The choice of $\alpha=0$, $\beta=1$ and $\gamma=0$ leads, for example, to the homogeneous Neumann boundary condition:

38 $$\nabla_{n}s=0\;,$$

which means no import of $s$ at the boundary $\Gamma$. In other words, Eq. (38) describes closed borders to surrounding countries outside $\Omega$. The diffusion coefficient function, $D:\Omega\to\mathbb{R}$, is responsible for the intensity or velocity of the diffusion process. From fluid or gas dynamics one knows [20]:

39 $$D=\frac{2}{3}\bar{v}\lambda\,,$$

with the averaged particle velocity, $\bar{v}$, and the mean free path, $\lambda$. The application of this ansatz to the movement of people in certain areas requires some assumptions for $\bar{v}$ and $\lambda$. The discussion of the mean distance of people in a certain federal state considering the means distances of homogeneous distributed people leads to the relation, $\lambda=\sqrt{A/N}$, with the area, $A$, and a number of inhabitants, $N$, for the relevant federal state. Let us assume the velocity $\bar{v}$ spanning 50 to 100 km/day, which is a gauge of mobility [1, 7]. Another suggesting heuristic is given with the assumption of $D$ assumed to be proportional to the population. The first ansatz, based on Eq. (39), was applied in the simulations with $\bar{v}=100$ km/day. However, these approaches looks to be a coarse approximation of such diffusion processes. As soon as the population (areas and number of inhabitants) of the federal states of Germany are different, $D$ is expected to be a location-dependent non-constant function. This means that the diffusion phenomenon is supposed to be of a different intensity in the different federal states of Germany. The data given in Table 3 define the function $D$. For example, one finds $D=0.48875$ km${}^{2}$/day for Bavaria, and $D=0.64355$ km${}^{2}$/day for Saxony-Anhalt.

If there are no sources or sinks for $s$, i.e., $q=0$, and the borders are closed, which means for the boundary condition (38), the initial boundary value problem of Eqs. (35), (36) and (38) has the steady-state solution:

40 $$s_{\text{st}}=\frac{\int_{\Omega}s_{0}(x)\,dx}{\int_{\Omega}dx}=\text{const.}$$

This is easy to verify, and this property is a characteristic of diffusion processes tending to equilibrium. It is quite complicated to model the source-sink function $q$ in an appropriate way. $q$ depends on the behavior of the population and the health policy of different federal states. Therefore, only very rough guesses can be made. It is known that people in Schleswig-Holstein are exemplary with respect to the recommendations to avoid infection with the COVID-19 virus which means $q<0$. On the other hand, in certain regions of Germany, people did not follow the indicated protocols, which means $q> 0$ existed for a long time (the government of Saxony has since changed the policy leading to $q<0$).

However, regardless of these uncertainties, one can obtain information about the pandemic propagation, for example, the influence of hotspots of high incidences (Saxony) to regions with low incidences (for example, South Brandenburg).

At the beginning of the year 2021 (January 14), the Robert Koch Institut (RKI), being responsible for the daily COVID-19 data collection, published the seven-day incidence data (of January 14, 2021 [14]), summarized in Table 3. The values of Table 3 are used as initial data for the function $s_{0}$ of Eq. (36).

The data in Table 3 [14] are used as a basis for the determination of the diffusion coefficient function.

**Table 3:** 7‑days incidence, people density [$/$km${}^{2}$], inhabitants $[/100\,000]$, area of the federal states of Germany [km${}^{2}$]

States	7‑days incidence	Density	Inhabitants	Area
Schleswig-Holstein	92	183	2904	15 804
Hamburg	115	2438	1847	755
Mecklenburg-West Pomerania	117	69	1608	23 295
Lower Saxony	100	167	7994	47 710
Brandenburg	212	85	2522	29 654
Berlin	180	4090	3669	891
Bremen	84	1629	681	419
Saxony-Anhalt	241	109	2195	20 454
Thuringia	310	132	2133	16 202
Saxony	292	221	4072	18 450
Bavaria	160	185	13 125	70 542
Baden-Wuerttemberg	133	310	11 100	35 784
North Rhine-Westphalia	131	526	17 947	34 112
Hesse	141	297	6288	21 116
Saarland	160	385	987	2571
Rhineland-Palatinate	122	206	4094	19 858
Munic	156	4700	1540	310

### The numerical solution of the initial boundary value problem (35), (36), (38)

Based on the subdivision of $\Omega$ (area of Germany) into finite rectangular cells $\omega_{j},\,j\in I_{\Omega}$, where $I_{\Omega}$ is the index set of the finite volume cells, and $\Omega=\cup_{j\in I_{\Omega}}\omega_{j}$, Eq. (35) was spatially discretized with a finite volume method. Along with the discrete boundary condition Eq. (38), one gets a semi-discrete system continuous in time

41 $$\frac{\partial s_{j}}{\partial t}=\nabla_{h}\cdot(D\nabla_{h}s_{j})+q_{j}\;,\;j\in I_{\Omega},$$

where $h$ indicates the discrete version of the $\nabla$-operator. The finite volume method is of a spatial order two; see, e.g., [39]. The time discretization is done with an implicit Euler scheme of order one. This allows us to work without strict restrictions for the choice of the discrete time-step $\Delta_{t}$. At each time level, one has to solve the linear equation system,

42 $$\frac{1}{\Delta_{t}}s_{j}^{n+1}-\nabla_{h}\cdot(D\nabla_{h}s_{j}^{n+1})=\frac{1}{\Delta_{t}}s_{j}^{n}+q_{j}\;,\;j\in I_{\Omega}\;,$$

for $n=0,\dots N,\,N=(T-t_{0})/\Delta_{t}$. $s_{j}^{0}$ was set to the incidence $s_{0}(x)$ for $x\in\omega_{j}$, $j=1,\dots,I_{\Omega}$.

Due to the complex geometry of the region $\Omega$ with the Jacobi method, an iterative solution method for Eq. (42) of the form $A\leavevmode\nobreak\ \boldsymbol{s}=\boldsymbol{b}$ was used. The coefficient matrix $A$ is irreducibly diagonal dominant, and therefore, the convergence of the Jacobi iteration method arises. For the discretization parameters, $\Delta_{t}$ values in the range of 0.1 to 1 day were chosen. The 2‑dimensional spatial discretization parameters $\Delta_{x}$ and $\Delta_{y}$ range from 7 to 14 km. With those discretization parameters, seven to twelve Jacobi-iterations are necessary to comply with the criterion (Euclidian norm of the relative error),

$$\frac{\left|\left|s^{n+1,i+1}-s^{n+1,i}\right|\right|_{2}}{\left|\left|s^{n+1,i}\right|\right|_{2}}<\epsilon$$

for $$\epsilon=10^{-4}$$.

### The qualitative behavior of the diffusion model

**Fig. 15:**
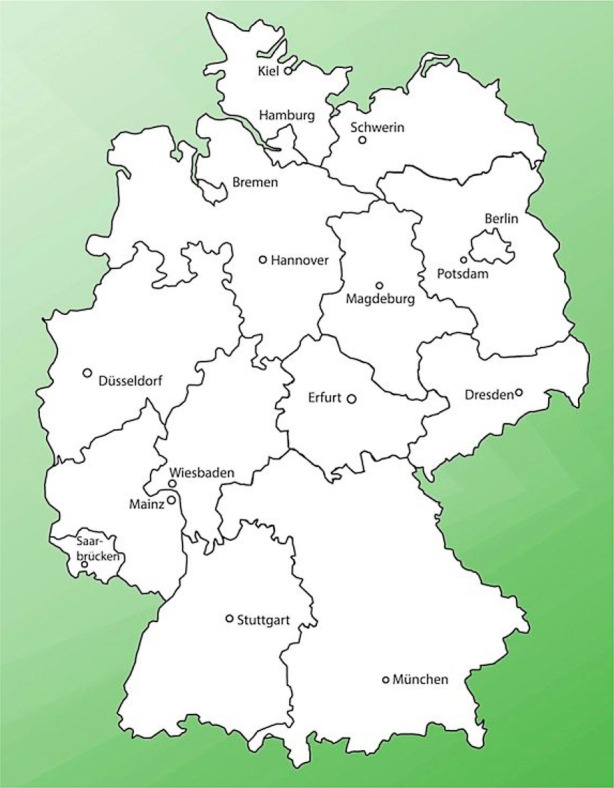
Map of Germany

**Fig. 16:**
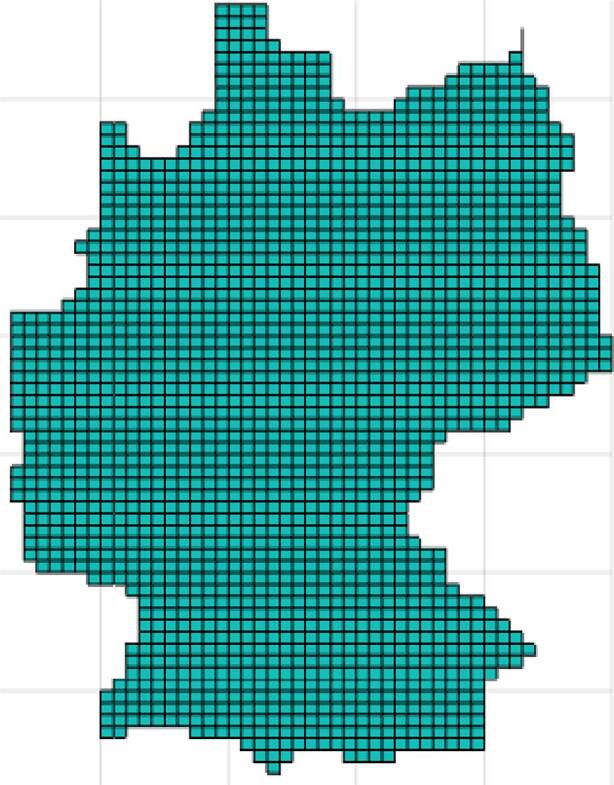
Rough contour of $\Omega$ and it’s discretization

Fig. 15 shows the map of Germany. For the sake of simplicity, the German border is approximated by a simple polygon. In Fig. 16, the region $\Omega$ is adumbrated, the size of the finite volume cells, $\Delta_{x}\times\Delta_{y}\approx(8\times 8)$ km${}^{2}$.

To validate the numerical method for the diffusion setting, a test should be made in order to reach a steady-state, i.e., the equilibrium given by Eq. (40). One can use large time-steps (of 10 days) as soon as there is no need to follow any time behavior here. With the seven-day-incidence of January 12, 2021 for the German federal states, gets the result $s\approx 160={\text{const.}}$ on $\Omega$. This is the constant value, which is evaluated using Eq. (40). It should be noted that this steady-state computation is only done for the validation reasons of the conservative approximation of the continuous mathematical model by the numerical finite-volume approximation. However, it is important to stress that a long-term simulation of more than a year is necessary to approach the steady-state. This is also a hint that the diffusion is a very slow, long-scale process.

**Fig. 17:**
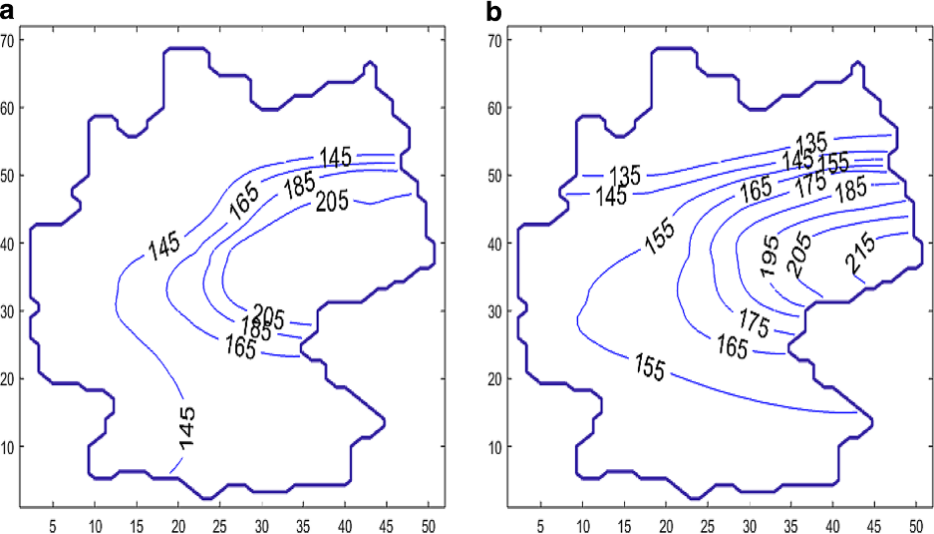
Contour lines of the seven-day incidence, $s$, at the time $t=15$ days **(a)** and $t=125$ days **(b)**

For the time-behavior simulations, let us start with the case $q=0$. $\Delta_{t}$ is then set to a half-day. Fig. 17 displays the initial state. The initial state is a piecewise constant function with values of the seven-day incidence of the 16 federal states where Munich is considered as a town with over a million inhabitants taken separately as it was excluded from Bavaria.

**Fig. 18:**
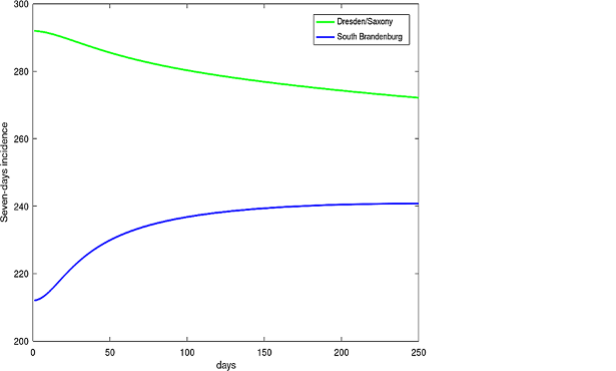
Time history of $s$ of Dresden (*upper line*) and Southern Brandenburg (*bottom line*)

Fig. 18 shows the development of the diffusion process with the change in contour lines of $s$ of the levels $135,155,175,195$, and 215 over a period of 100 days. Especially in the border regions (Saxony—Brandenburg, Saxony—Bavaria, Saxony—Thuringia), one can observe a transfer of incidence from the high level incidence of Saxony to the neighboring federal states. Furthermore, the high incidence level of Berlin was transferred to the nearby Brandenburg region. The northern states with a low incidence level were only influenced weakly by the other states. A typical smoothing and decreasing of the incidence gradients can also be observed. The short-horizon forecast confirms the qualitative development of the incidence in Germany. A finer resolution of the incidence propagation will be considered below by finer modeling of the source-sink term $q$.

In Fig. 18, the development of the seven-day incidence of a high incidence region (Dresden) compared to a low incidence region (South Brandenburg) is shown. With the parameters $\alpha$, $\beta$ and $\gamma$ of the boundary condition Eq. (3), it is possible to describe several situations at the borders of the boundary $\Gamma$ of $\Omega$. The case with $\alpha=0$, $\beta=-D$ and $\gamma\neq 0$ describes a flux through the border. Such a scenario is used in the following example to describe the journey home of people with COVID-19 from Austria to Bavaria.

The boundary condition at the border crossing reads:

$$-D\nabla s\cdot\vec{n}=\gamma\;.$$

**Fig. 19:**
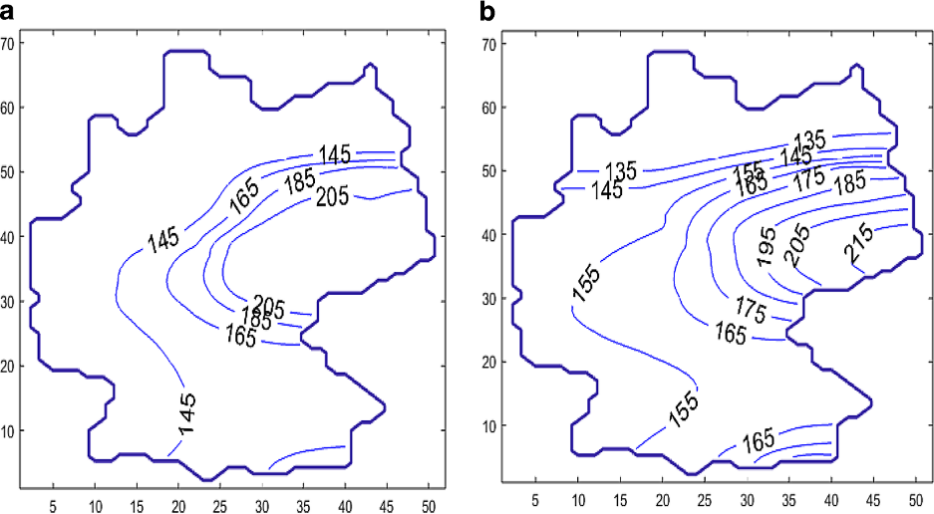
Contour lines of $s$ at $t=15$ days **(a)**, $t=125$ days **(b)**, with the source-sink function $q=0$, and the coefficient $\gamma=250$ km/day

The initial state $s_{0}$ the same as in the example above. $\gamma> 0$ means an “inflow” of people with COVID-19, $\gamma<0$ indicates a loss of people with COVID-19, while $\gamma=0$ refers to a closed border. In Fig. 19, the move of the contour lines of $s$ for the case $\gamma=250$ km/day is shown.

At the southern border of Bavaria, one can observe the increase of $s$ caused by the flux of $s$ from Austria to Bavaria.

The results without a source-sink-term ($q=0$) describe the qualitative trend, which was observed in the pandemic development. To describe the whole pandemic process of long-scale diffusion and the small-scale local virus transmission, it is necessary to consider local epidemic spreading models, as it is done in the next section.

### Consideration of the local transmission via a *SIR* model in the diffusion model

The previous section demonstrates the diffusion as a long-scale process. On the other hand, a small-scale process occurs with the direct virus transmission via epidemiological infection. This process can be described with a *SIR* model, for example.

The change of $s$ per day can be divided into a part coming from diffusion and another part coming from the local transmission of the virus. The second issue will be modeled with the *SIR* model. The local virus transmission means, in other words, the consideration of a *SIR* model in the federal states of Germany separately. The *SIR* model is defined by the following system of equations (see, e.g., [26, 29]):

43 $$\begin{aligned}\frac{dS_{j}}{dt} & = & -\kappa_{j}\frac{I_{j}}{N_{j}}S_{j}\;,\end{aligned}$$

44 $$\begin{aligned}\frac{dI_{j}}{dt} & = & \kappa_{j}\frac{I_{j}}{N_{j}}S_{j}-\eta_{j}I_{j}\;,\end{aligned}$$

45 $$\begin{aligned}\frac{dR_{j}}{dt} & = & \eta_{j}I_{j}\;,\end{aligned}$$

where $j$ defines the respective federal state, and $S_{j}$, $I_{j}$ and $R_{j}$ are the groups of susceptible, infected and removed people.

$N_{j}$ is the population of the respective federal state. $\eta$ is the reciprocal value of the typical time from infection to recovery ($\eta=1/14\approx 0.07$). $\kappa_{j}$ is the average number of contacts per person per time multiplied by the probability of disease transmission for a contact between a susceptible and an infectious subject.

The relation between the actual reproduction number, ${\cal R}$, and $\kappa$ and $\eta$ is ${\cal R}={\kappa}/{\eta}$.

To clarify a possible relation between actual non-pharmaceutical measures of the government and the values of $\kappa$ (or ${\cal R}$), let us consider the development of people with COVID-19 in the period from November 18, 2020 to April 24, 2021.

**Fig. 20:**
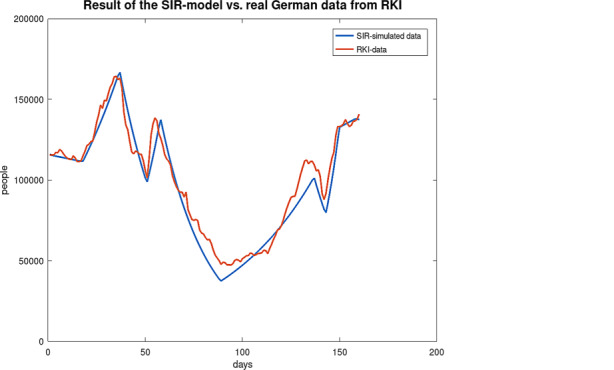
Comparison of real data [14] and simulation results

Fig. 20 shows the RKI data [14] and the result of the simulation with the *SIR* model. The curve shows the implication of the drastic changes of the measures taken by the politicians with a sequence of local minimums followed by local maximums. The first local minimum seen was reached on December 5, 2021, the first local maximum found on December 24, 2021, the next minimum found on January 7, 2021, and the next local maximum on January 14, 2021. In Table 4, the possible values of $\kappa$ to obtain the curve of the people with COVID-19 for the simulation with the *SIR* model are shown. The possible $\kappa$-values for the chronological periods are obtained with a simple trial-and-error method.

**Table 4:** Simulated period-dependent average number of contacts per person per time, $\kappa$, and actual reproduction number, $\cal R$, for Germany.

Period	$\kappa$	${\cal R}$
November 18, 2020–November 23, 2020	0.068	0.97143
November 24, 2020–January 6, 2021	0.92	1.3143
January 7, 2021–January 13, 2021	0.12	1.7143
January 14, 2021–February 13, 2021	0.028	0.4
February 14, 2021–April 2, 2021	0.092	1.3143
April 3, 2021–April 8, 2021	0.028	0.4
April 9, 2021–April 15, 2021	0.1464	2.0914
April 16, 2021–April 24, 2021	0.076	1.0857

The measures taken by the government from the end of April 2021 can be compared with the measures for the period beginning at January 14 with a $\kappa$-value of 0.028. For the propagation of the pandemic from April 25, this $\kappa$-value is used. Due to the fact that the government measures are based on the infection control law, which are valid from April 25, 2021, the same $\kappa$-value is used for all federal states of Germany.

In what follows, the diffusion model (35), (36), (38) coupled with the *SIR* model (43)–(45) is used.

To take into account the long-scale and small-scale processes, one considers, after the diffusion steps with the size $\Delta_{t}$, the model (43)–(45) for a time-interval $\Delta_{t}$. This means to solve a family of initial value problems in the interval $[t_{p},t_{p}+\Delta_{t}]$ in every diffusion step of Eq. (42) from $t_{p}$ to $t_{p}+\Delta_{t}$ (see Algorithm 1).The initial values for $I_{j}(t_{p})$ are used as the mean values of $s$ (Table 3) of the respective federal states. The first values of $R_{j}(t_{p})$ (in the first diffusion step) are set to zero and the $S_{j}(t_{p})$ values come from the relation $N_{j}=S_{j}+I_{j}+R_{j}$. The result of the initial value problem $I_{j}(t_{p}+\Delta_{t})$ is converted to $s_{j}(t_{p}+\Delta_{t})$ and used to determine $q$ for Eq. (42) by the changing rate of $s_{j}$, which means

$$q(x,t_{p}+\Delta_{t})=\frac{s_{j}(t_{p}+\Delta_{t})-s_{j}(t_{p})}{\Delta_{t}}\;,\;\;x\in\omega_{j}\;,$$

during the time $\Delta_{t}$, caused by the process modeled with the equation system (43)–(45). In the deterministic case ($\nu=0$), the Euler method is used to solve the initial value problem per diffusion time-step (with a time-step of $\delta_{t}=\Delta_{t}/10$). For constant coefficients $\kappa$, $\eta$ there are possibilities of finding analytic solutions of the *SIR* system, which can be found in [28, 42]. However, for time-dependent coefficients, numerical methods have been used to find a solution. Here, it is important to note that the step-sizes $\Delta_{t}$ and $\delta_{t}$ used are chosen heuristically. Let us note that the analysis of the physics of time-scales of both the local transmission and the diffusion process is an interesting point and should be considered in further investigations of such combined modeling together with the parameters of the diffusion process; see, e.g., [18].


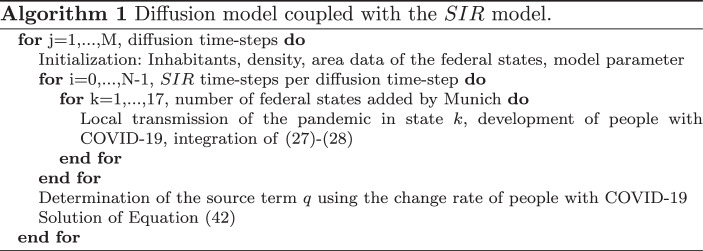


**Fig. 21:**
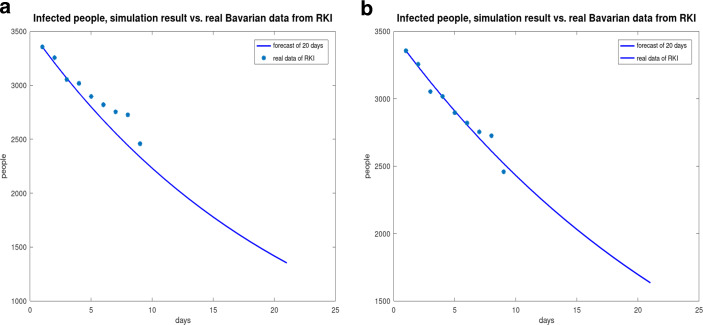
Course of people with COVID-19 in Bavaria, from April 26, 2021 to May 5, 2021 without diffusion (**a**) and with diffusion (**b**)

Due to the poor informativeness of surface graphs and contour lines, the propagation of the people with COVID-19 in Bavaria is used to compare the simulated results with the real data of the RKI. The result of the diffusion model coupled with the *SIR* model over the period from April 26 to May 5 is shown in Fig. 21 ($\Delta_{t}=1$ day, $\delta_{t}=\Delta_{t}/10$). In addition, to the nine days where the real data are known, a forecast up to May 16, 2021 is made.

**Fig. 22:**
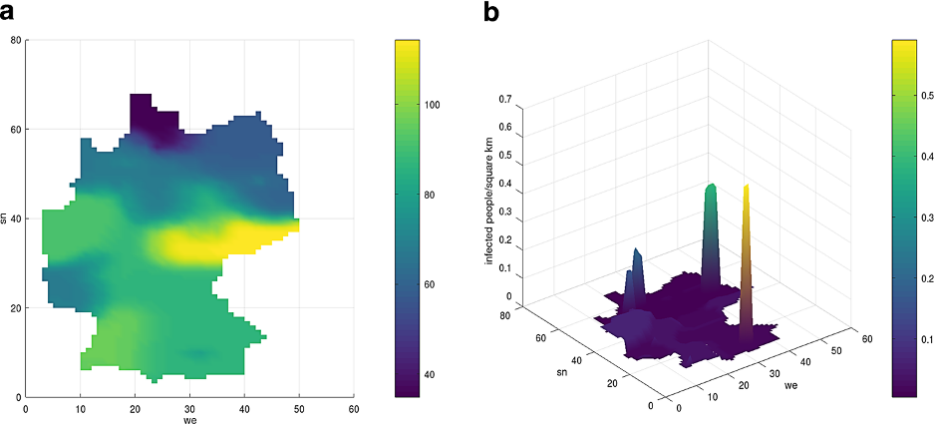
Forecast of the seven-day incidence, $s$, **(a)**, and distribution of people with COVID-19 **(b)** after 20 days, for the *SIR* model coupled with the diffusion concept

For the reinterpretation (by counting back using the population and the area of the federal states) of the result for the seven-day incidence, in Fig. 22 the incidence with the congruous distribution of the people with COVID-19 per square kilometer is considered. It is obvious that the people with COVID-19 are concentrated in the congested urban and metropolitan areas such as Munich, Hamburg, Berlin, and Ruhr.

Sect. 9 is a recapitulation of paper [10]. It should be mentioned that these thoughts about the term “diffusion” are still under consideration and will be an important issue of further research.

## Discussion and Conclusions

In this paper, we reviewed some basic properties of the *SIR* model to describe the progression of the COVID-19 pandemic. Further it demonstrated the *SIR* model and some modifications by numerical simulations of the German pandemic situation. It was found that the timing of the lockdown is crucial in the progression of a pandemic. It could be shown that a very early start of limited social distancing measures for a period of $\Delta t$ days leads only to a displacement of the climax of the pandemic, but not really to an efficient flattening of the curve of the number of infected people.

The intervention measures are more efficient, and one can observe a descent in the number of infected people if the social distancing is started beyond the dynamical lockdown time $t_{0}$. However, in this case, a second bump of the curve of infected people will also occur. A stepwise return to normality turned out to be the most efficient way to overcome the climax of a pandemic.

For the calibration of the *SIR* model, i.e., the evaluation of the parameter $\beta$, the non-linear regression comes up with significantly better results than the log–linear regression. This is evident with the comparison of the graphs of the evaluated exponential functions.

It must be noted again that the parameters $\beta$ and $\kappa$ were guessed very roughly. Depending on the capabilities and performance of the health systems and further research results and experiences of the respective countries, those parameters may look different.

**Fig. 23:**
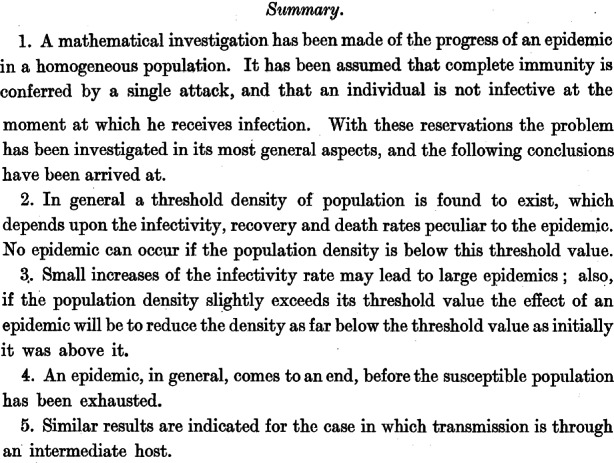
Original conclusions, facsimile of [26]

The conclusions to control the pandemic of today are not really different to those of Kermack/McKendrick, which are cited in Fig. 23. The contacts of people must be reduced, the personal and social environment must be efficiently sanitized and the vulnerable people must be eminently protected. The vaccination/immunization of the population is the most important issue to reach the herd immunity which leads to a decreasing number of infected people and terminates the pandemic in the end by switching to an endemic situation.

It has to point to the second bump in the progression of the number of infected people as an important issue of limited lockdowns. This must be taken into account in all decisions made by physicians and politicians in connection with the handling of the pandemic.

Finally, it must be said that the *SIR* models and their modifications are valuable instruments to describe the pandemic and forecast the infection history over short periods. But it is necessary to adjust the model and its parameters depending on the respective local situation. Due to the supposition of homogeneity of people distribution, density, behavior and so on the *SIR*-model provides mean propositions and forecast the infection history over short periods. But it is necessary to adjust the model and its parameters depending on the respective local situation. For a finer local resolution of the pandemic propagation one can consider separate considerations for smaller regions rather than whole countries like Germany or Spain. This was considered be a recapitulation of [10] with separate models for example for Bavaria, Saxonia, Thuringia combined with the model of diffusion processes.

In conclusion, it must be said that the results of the simulations using the *SIR* model describe, in a way, the worst case. A lot of interventions made by politicians and physicians can disturb the progression of the pandemic in a positive way. However, not all measures and interventions can be described by *SIR*-type models. This allows the conjecture that the real pandemic will be weaker than the simulation results of the model.

Due to the development of the data concerning infected people, which is less dramatic than the forecast of the mathematical modeling community, a bashing of science, mainly physicists, mathematicians and virologists emerges. This is regrettable, but “There is no glory in prevention” – a well-known saying of physicians – and we must live with the criticism in politics and the printed and electronic media. But then without respectable warnings about the risks of the COVID-19 virus aggressiveness by the named scientists a disaster would have been ineluctable.

## Appendix

### German data for the estimation of $\beta$

In the following table the German data of infected people from February 13 to March 19, 2020 is listed.

**Table:**

Day	Month	Infected people
1.30000000e+01	2.00000000e+00	1.50000000e+01
1.40000000e+01	2.00000000e+00	1.50000000e+01
1.50000000e+01	2.00000000e+00	1.50000000e+01
1.60000000e+01	2.00000000e+00	1.50000000e+01
1.70000000e+01	2.00000000e+00	1.50000000e+01
1.80000000e+01	2.00000000e+00	1.50000000e+01
1.90000000e+01	2.00000000e+00	1.50000000e+01
2.00000000e+01	2.00000000e+00	1.50000000e+01
2.10000000e+01	2.00000000e+00	1.50000000e+01
2.20000000e+01	2.00000000e+00	1.50000000e+01
2.30000000e+01	2.00000000e+00	1.50000000e+01
2.40000000e+01	2.00000000e+00	1.50000000e+01
2.50000000e+01	2.00000000e+00	1.50000000e+01
2.60000000e+01	2.00000000e+00	1.70000000e+01
2.70000000e+01	2.00000000e+00	2.10000000e+01
2.80000000e+01	2.00000000e+00	4.70000000e+01
2.90000000e+01	2.00000000e+00	5.70000000e+01
1.00000000e+00	3.00000000e+00	1.11000000e+02
2.00000000e+00	3.00000000e+00	1.29000000e+02
3.00000000e+00	3.00000000e+00	1.57000000e+02
4.00000000e+00	3.00000000e+00	1.96000000e+02
5.00000000e+00	3.00000000e+00	2.62000000e+02
6.00000000e+00	3.00000000e+00	4.00000000e+02
7.00000000e+00	3.00000000e+00	6.84000000e+02
8.00000000e+00	3.00000000e+00	8.47000000e+02
9.00000000e+00	3.00000000e+00	9.02000000e+02
1.00000000e+01	3.00000000e+00	1.13900000e+03
1.10000000e+01	3.00000000e+00	1.29600000e+03
1.20000000e+01	3.00000000e+00	1.56700000e+03
1.30000000e+01	3.00000000e+00	2.36900000e+03
1.40000000e+01	3.00000000e+00	3.06200000e+03
1.50000000e+01	3.00000000e+00	3.79500000e+03
1.60000000e+01	3.00000000e+00	4.83800000e+03
1.70000000e+01	3.00000000e+00	6.01200000e+03
1.80000000e+01	3.00000000e+00	7.15600000e+03
1.90000000e+01	3.00000000e+00	8.19800000e+03

It is recommended to evaluate the data of this table to empathize the estimation of the parameter $\beta$ by the root mean square approximation method which was carried out above.

### Approximated analytic solution of the *SIR* model

Above with

$$R(t)=\frac{\rho^{2}}{S_{0}}\left(\left(\frac{S_{0}}{\rho}-1\right)+\alpha\tanh\left(\frac{\alpha\gamma t}{2}-\phi\right)\right)$$

was given an approximation of the development of removed people by the exact solution of the Riccati-equation (10), and with

$$I(t)=\frac{\alpha^{2}\rho^{2}}{2S_{0}}\text{sech}^{2}\left(\frac{\alpha\gamma t}{2}-\phi\right)\;$$

the consequence for the compartment $I$. Maybe it is interesting to evaluate these formulas for appropriate data of the actual pandemic by eligible graphics software (python, MATLAB etc., $\alpha$ and $\rho$ are defined above) as an exercise of the useful application of computer-algebra systems. A second exercise is the proof of $R(t)$ to be a solution of the Riccati equation (10).

## Footnotes

1 The term “recovered” includes not only the people who are fully recovered, but the people who are exited or removed of the pandemic process also, for example the dead people.

2 To grasp super-spreader events it is necessary to apply the *SIR* model on the relevant cluster in a relevant sub-region.

3 The compartment $R$ includes the people who passed away.

4 We suppose here a population of 70 million because not all members of the 83 million German population are affected by the pandemic. But this assumption does not change the principal trend.

5 The practical application of the model and its numerical treatment yields appropriate solutions as a good approximation of the modeled problem of the pandemic.

6 $\text{sech}=1/\cosh$ is the secans hyperbolicus function, which we get with the differentiation of the $\tanh$-function.

7 Brownian motion, is the random motion of particles suspended in a medium (a liquid or a gas). This pattern of motion typically consists of random fluctuations in a particle’s position inside a fluid. This motion is named after the botanist Robert Brown, who first described the phenomenon in 1827 in biology.

8 For a better readability we leave out the index $t$.

9 Due to the property of $S,\,I$, to be non-negative, the matrix $C$ is at least positive semi-definite and symmetric. Therefore, the square root exists and the Cholesky decomposition exists for the positive definite case.

10 I will understand 20% of normality by a lockdown, this means $\beta=0.2\beta_{0}$.
